# Breast adipose tissue macrophages (BATMs) have a stronger correlation with breast cancer survival than breast tumor stroma macrophages (BTSMs)

**DOI:** 10.1186/s13058-021-01422-x

**Published:** 2021-04-13

**Authors:** Lili Lin, Christina Kuhn, Nina Ditsch, Thomas Kolben, Bastian Czogalla, Susanne Beyer, Fabian Trillsch, Elisa Schmoeckel, Doris Mayr, Sven Mahner, Udo Jeschke, Anna Hester

**Affiliations:** 1 Department of Obstetrics and Gynecology, University Hospital, LMU Munich, Marchioninistr. 15, 81377 Munich, Germany; 2 Department of Obstetrics and Gynaecology, University Hospital Augsburg, Stenglinstr. 2, 86156 Augsburg, Germany; 3 Department of Pathology, LMU Munich, Thalkirchner Straße 36, 80337 Munich, Germany

**Keywords:** Breast cancer, Macrophages, Adipocytes, Adipose tissue, Prognostic marker, EP3

## Abstract

**Background:**

An abundance of tumor-associated macrophages has been shown to be an independent prognostic factor for a poor prognosis of human breast cancer (BC). Adipose tissue accounts for the largest proportion of the breast and has also been identified as an independent indicator of poor survival in BC. This study aims to elucidate if the influence of adipose tissue in BC might be mediated by macrophages. The roles of macrophages in the breast tumor-stroma (breast tumor stroma macrophages, BTSM) and macrophages in the surrounding adipose tissue (breast adipose tissue macrophages, BATM) were explored separately.

**Methods:**

Two hundred ninety-eight BC tissue samples were analyzed immunohistochemically. The number of macrophages was detected by CD68+ staining. The quantity of BATMs and BTSMs was correlated to clinical and pathological parameters as well as to disease-free survival (DFS) and overall survival (OS).

**Results:**

The amounts of BATMs and BTSMs strongly correlated with each other (*r* = 0.5, *p* = 2.98E−15). The quantity of BTSMs, but not of BATMs, was significantly associated with the BC molecular subtype (*p* = 0.000011), and all triple-negative BC tumors contained high amounts of BTSMs. BATMs were negatively associated with DFS (*p* = 0.0332). Both BATMs (*p* = 0.000401) and BTSMs (*p* = 0.021) were negatively associated with OS in the Kaplan-Meier analysis, but only BATMs remained an independent factor in the multivariate Cox-regression analysis (HR = 4.464, *p* = 0.004). Combining prostaglandin E2 receptor 3 (EP3)-expression and the quantity of BATMs, a subgroup with an extremely poor prognosis could be identified (median OS 2.31 years in the “high BATMs/low EP3” subgroup compared to 11.42 years in the most favorable “low BATMs/high EP3” subgroup, *p* = 0.000002).

**Conclusion:**

Our findings suggest that BTSMs and BATMs seem to be involved differently in BC. Breast adipose tissue might contribute to the aggressiveness of BC via BATMs, which were independently associated with BC survival. BATMs’ role and occurrence might be functionally dependent on EP3, as a combination of both factors was strongly associated with survival. Targeting BATMs—eventually in combination with targeting the EP3-pathway—might be promising for future therapies.

**Supplementary Information:**

The online version contains supplementary material available at 10.1186/s13058-021-01422-x.

## Background

Macrophages play an important role in regulating the migration and invasion of breast cancer (BC) cells and in promoting BC metastasis [1]. Clinical studies have shown that the abundance of tumor-associated macrophages (TAMs) in BC tissue is an independent prognostic factor for a poor prognosis: high levels of TAMs in BC were associated with an impaired disease-free survival (DFS) and overall survival (OS) [2, 3]. Macrophages can be subdivided according to their phenotype into the two subgroups of M1 (“classically activated”) and M2 (“alternatively activated”) macrophages [4–7]. M1 macrophages are usually activated by monocytes due to their induction by bacteria or its product lipopolysaccharide (LPS). M1 macrophages show a high antigen presentation ability and high secretion levels of interleukin-12 (IL-12) [4–8]. M2 macrophages can be activated by monocytes due to their induction by interleukin-4 (IL-4), interleukin-6 (IL-6), macrophage colony-stimulating factor (MCSF), or prostaglandin E2 (PGE2) [4–7, 9, 10]. Initially, M1 macrophages were characterized as pro-inflammatory, while M2 macrophages were described to regulate the repair of tissues and the resolution phase of inflammation [11]. In the case of immune homeostasis, the two sub-populations are assumed to be in equilibrium. Today, it is well known that their functions show wider overlapping [12, 13]. In immunohistochemical staining, macrophages can be detected by specific CD molecules. CD68 is the most common marker for monocytes and macrophages independent of their polarization [14–16]. M2 macrophages can be detected in tissues using the specific marker CD163 [14, 15, 17]. In BC, high levels of tumor infiltrating CD163+ macrophages have been associated with higher proliferation rates, lower tumor cell differentiation, and a lack of hormone receptor (HR) expression (HR negativity) [18]. Additionally, more CD163+ macrophages could be detected in triple-negative breast cancer (TNBC) than in other BC subtypes [19, 20]. PGE2, which contributes to M2 polarization, is a tissue hormone with various effects that also exerts direct effects on tumor cells. The role of PGE2 and its receptors prostaglandin E2 receptor 1-4 (EP 1-4) has been widely investigated in BC [21, 22]. EP2 and EP4 are the best evaluated receptors and are mainly assumed to be negative prognostic factors [21, 23], while EP1 and EP3 are less well understood and have shown tumor-suppressive effects [24, 25].

TNBC is the biological subgroup of BC lacking the expression of the HRs, estrogen receptor (ER), and progesterone receptor (PR) and not showing an amplification of human epidermal growth factor receptor 2 (HER2) [26]. TNBC represents approximately 15–20% of all BC cases [27] and is considered to be more aggressive, showing a poorer prognosis and more often visceral metastases than other subtypes of BC [28]. As the classical target structures are missing, no targeted therapies are available in TNBC so far. Only recently, a milestone in TNBC therapy has been achieved through the demonstration of the beneficial effect of immune therapies [29].

The largest proportion of breast tissue consists of adipose tissue [30]. Recent studies have shown that adipose tissue from grafts can potentially promote or accelerate the development or local recurrence of subclinical breast tumors [31]. Zhu et al. [32] summarized the association of adipose cells and BC cells as follows: (i) infiltrating BC cells can greatly affect the surrounding adipose cells, (ii) adipose cells around the tumor show a modified phenotype and specific biological characteristics, and (iii) surrounding adipose cells modify BC cells characteristics and their phenotype leading to a more aggressive behavior. Even after adjusting for body mass index (BMI), age, and menopausal status, fatty breasts (very low density, VLD) are still an independent indicator of poor survival in BC [33]. Furthermore, BC in fatty breasts usually contains high levels of M2-like macrophages, which might reduce local inflammation, contribute to tumor promotion, and lead to an impaired survival [33]. However, the localization (in the tumor or in the tumor surroundings) and the exact phenotype of these macrophages have not yet been fully clarified.

A further feature found in adipose tissue, which is considered to be a hallmark of the proinflammatory process in adipose tissue, are the so-called crown-like structures (CLS, recently reviewed by Faria et.al [34]) CLS consist of hypertrophied, necrotic adipocytes (that need to be resorbed) surrounded by adipose tissue M1 macrophages [35–38]. CLS might promote BC and might contribute to the fact that obese women are more likely to be diagnosed with larger and higher-grade BC and have higher incidence of metastases than lean individuals [39]. In obese and overweight patients, more adipose cell death (represented by a higher number of CLS) resulting in a release of free fatty acids occurs, and an infiltration of pro-inflammatory M1 macrophages maintaining the exacerbated inflammatory state has been shown [40]. In adipose tissue of lean individuals, M2 predominate M1 macrophages (M2:M1 ratio 4:1), while in obese individuals, much more M1 than M2 macrophages can be detected (M2:M1 ratio 24:65) [41–43]. An increased number of CLS has been shown in breast adipose tissue from especially obese BC patients, which has been related to high recurrence rates and poor survival [44].

However, what makes breast adipose tissue contributing to BC promoting events—not only in obese BC patients but probably also in lean individuals—remains an intriguing question to be solved. The data mentioned above indicates that the quantity of macrophages in the adipose tissue of BC might be important. This study aims to elucidate the role of adipose tissue in BC development in general. Therefore, the association between adipocytes, macrophages, and BC cells was evaluated in an unselected cohort of primary BC patients, independent of a pre-set condition of overweight or obesity. Furthermore, to clarify the role of the localization of the macrophages, we analyzed macrophages infiltrating in the breast adipose tissue (breast adipose tissue macrophages, BATMs) and in the breast tumor-stroma tissue (breast tissue stroma macrophages, BTSMs) separately. The quantity of BATMs and BTSMs was correlated to clinical and pathological parameters as well as to survival.

## Methods

### Human tissue samples

In this study, 320 consecutive patients who underwent surgery for BC from 2000 to 2002 at the Department of Gynecology and Obstetrics, Ludwig-Maximillian’s-University of Munich, Germany, and of whom tumor tissue was still available were primarily included. To diagnose BC, all patients had undergone tumor biopsy prior to surgery for BC but no patient has undergone any other prior treatment. In the further analyses, only cases with a diagnosis of sporadic BC and without family history for BC were included (*n* = 306). Patients with primary distant metastases (*n* = 6) and patients with only ductal carcinoma in Situ (DCIS) but without invasive BC (*n* = 2) were excluded from further analyses. So, 298 patients were included in the final analyses. The Institute of Pathology, Ludwig-Maximillian’s-University of Munich, assigned the tumor grading (according to the Elston-Ellis system); tumors were classified according to the American Joint Committee on Cancer (AJCC) TNM staging system. The surrogate intrinsic BC subtype (the following five groups) was defined by immunohistochemistry: luminal A-like (ER/PR positive, HER2 unamplified, ki67 less than 14%), luminal B-like (ER/PR positive, HER2 unamplified, ki67 more than 14%), basal-like/triple negative (ER and PR negative, HER2 unamplified), HER2 amplified luminal-like (ER/PR positive, HER2 amplified), and HER2 amplified non-luminal-like (ER/PR negative, HER2 amplified). We further included the expression of EP3 (measured immunohistochemically and quantified by the immune reactive score, IRS), which has already been performed by our team previously [24] as well as further prognostic factors previously described by our group into the analysis. Patient data regarding patient age, HR status, HER2-amplification, metastasis, local recurrence, progression, and survival were retrieved from the Munich Cancer Registry. DFS and OS were statistically analyzed after an observation period of up to 12 years.

### Immunohistochemistry

Formalin-fixed tissue slides were embedded in paraffin wax for immunohistochemistry. The samples were de-paraffinized in xylol for 20 min and rinsed in 100% ethanol. Methanol/H_2_O_2_ incubation for 20 min was performed to inhibit endogenous peroxidase reaction. Afterwards, the specimens were rehydrated in a descending alcohol gradient, starting with 100% ethanol and ending with distilled water. The samples were cooked in a pressure cooker, containing a sodium citrate buffer (pH = 6.0), which consisted of 0.1 mM citric acid and 0.1 mM sodium citrate in distilled water. Subsequently, the samples were washed in PBS twice and incubated with a blocking solution (reagent 1, ZytoChem Plus HRP Polymer System (Mouse/Rabbit), Zytomed, Berlin, Germany) for 5 min. Then, an incubation with the primary antibody was performed with each section for 16 h at 4 °C. Primary anti-CD68-antibody (Rabbit IgG polyclonal, Sigma Aldrich, St. Louis, MO, USA) was used for tissue slides staining. Following every subsequent step, the samples were washed twice in PBS (pH = 7.4). “Post block” (reagent 2) for 20 min and HRP-Polymer (reagent 3) for 30 min were applied. The chromogen-substrate staining was carried out using the Liquid DAB+ Substrate Chromogen System (Dako Scientific, Glostrup, Denmark) for 2 min. The reaction was stopped by applying distilled water. Finally, the tissue samples were counterstained with hemalum for 2 min and blued in tap water. The specimens were dehydrated in an ascending alcohol gradient and cover slipped with Eukitt® quick hardening mounting medium (Sigma Aldrich, St. Louis, MO, USA). Placenta tissue served as positive control and negative control. All slides were analyzed using the microscope Leitz Wetzlar (Wetzlar, Germany; Type 307-148.001 514686).

### Quantity of macrophages

**Fig. 1:**
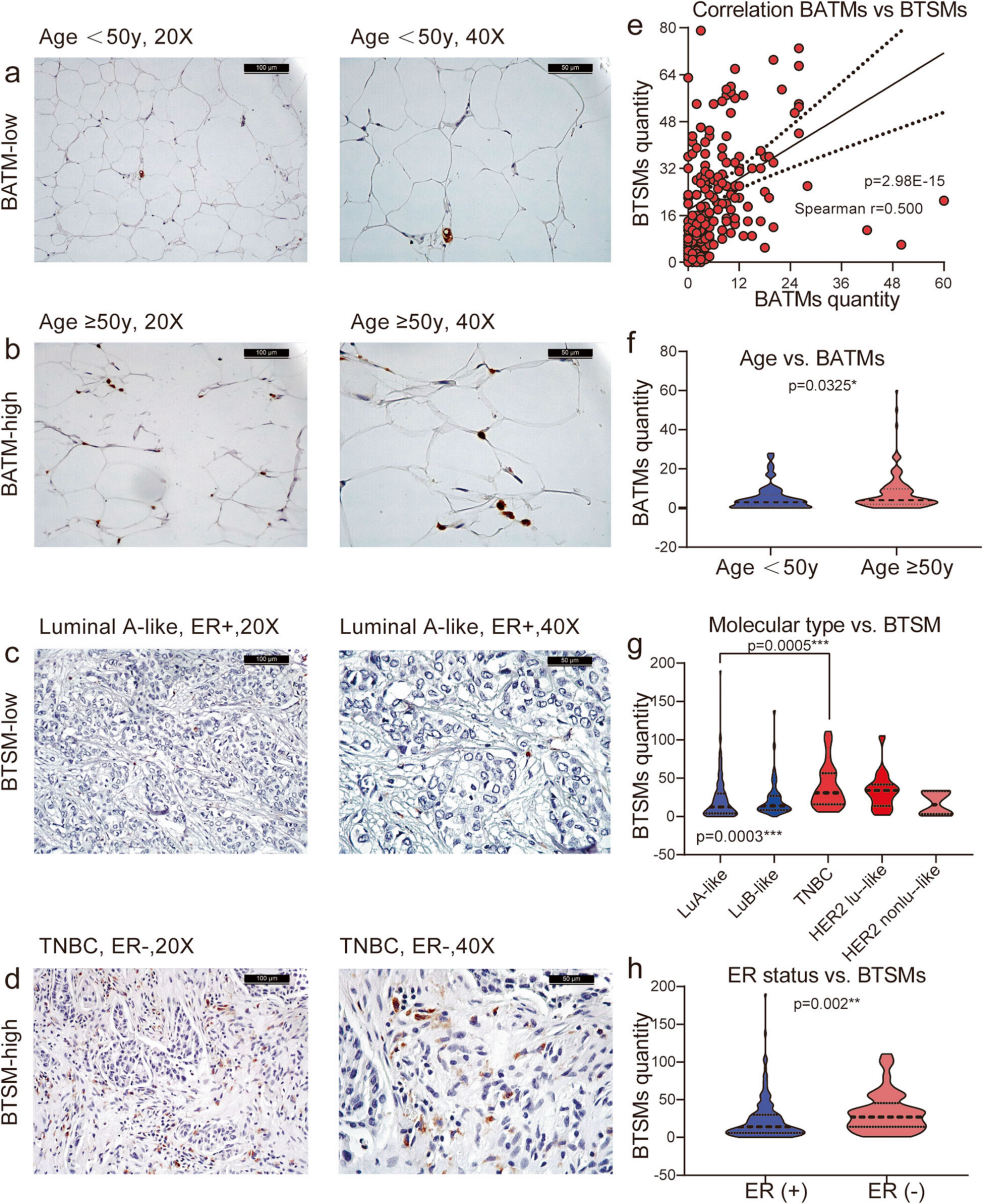
BATMs and BTSMs distribution in BC. A strong correlation was found between the quantity of BATMs and BTSMs (*r* = 0.5, *p* = 2.98E−15) (**e**). The number of BATMs in patients aged older than 50 years was significantly higher than in patients younger than50 years (**a**, **b**, **f**). The quantity of BTSMs was significantly related to BC molecular type (*p* = 0.0003, **g**) and was significantly higher in TNBC than in luminal A-like BC (*p* = 0.0005, **g**) (**c**, **d**). Regarding only ER-status independent of the biological subtype, the quantity of BTSM in ER- patients was significantly higher than in ER+ patients (*p* = 0.002, **h**) (**c**, **d**). BTAMs, breast adipose tissue macrophages; BTSMs, breast tumor-stroma macrophages; ER, estrogen receptor; HER2, human epidermal growth factor receptor 2; LuA-like, luminal A-like; LuB-like, luminal B-like; TNBC, triple-negative breast cancer; HER2 Lu-like, HER2 amplified luminal-like; HER2 nonLu like, HER2 amplified non luminal-like

CD68 positivity is an indicator for all macrophages [33]. The immunohistochemical staining for macrophages was performed as described above. Two hundred ninety-eight adequately CD68-immunostained tissue sections were available for analysis. CD68 is located in the cytoplasm of the macrophages; the positive staining is brownish-yellow or brown particles (Fig. 1a–d). The staining intensity of CD68 in the BC tissue samples was limited to the number of macrophages at each respective site. Cancer cells did not express CD68. The tumor cells which were pleomorphic and atypical with large nuclei and nucleoli were easy to distinguish from the macrophages. Other cells like fibrocytes and adipocytes were also not stained with CD68. Therefore, we directly counted the CD68 positive cells and from there concluded the number of macrophages. Three investigators counted the number of macrophages in four views of each IHC slide, separately in the breast tumor-stroma section and the breast adipose tissue section. The distance between this two analyzed areas in each slide is between 400 μm–600 μm. We distinguished and excluded necrotic areas and areas with high mitotic activity when analyzed the macrophage in the breast tumor-stroma section. The average value represented the quantity of macrophages in the respective sections. The macrophages in the breast tumor-stroma section were defined as breast tumor-stroma macrophages (BTSMs); the macrophages in the adipose tissue around the tumor were named breast adipose tissue macrophages (BATMs). The levels of BTSMs and BATMs were categorized as either low or high and the resulting groups were named “BTSM/BATM-high” and “BTSM/BATM-low”. The cutoff values for the categorization were determined using receiver operating characteristic curve (ROC-curve) analysis based on OS and DFS. ROC analysis is commonly used to measure the diagnostic accuracy of a biomarker and uses the area under the ROC curve (AUC). This method defines the optimal cutoff value as the value at which sensitivity and specificity are closest to the AUC and at which the absolute value of the difference between sensitivity and specificity is minimal. Slides with a BATMs quantity ≤ 9.5 were defined as “BATM-low” and a BATMs quantity > 95 was defined as “BATM-high”. A BTSMs quantity ≤ 4.5 was defined as “BTSM-low” and a BTSMs quantity > 4.5 was defined as “BTSM-high.” Both parameters, the total quantity of BTSMs and BATMs as continuous variables, as well as the categorized variables “BATMs/BTSM-high” and “BATMs/BTSM-low” were compared to known clinical and pathological parameters and further prognostic factors previously determined in this collective by our group. Only the categorized variables were used to analyze the influence of BATMs and BTSMs on OS or DFS.

### Statistics

IBM SPSS software version 26 was used to analyze data. Microsoft Excel 2017 was used for illustrations. *P* values < 0.05 were considered statistically significant. Chi square test, Mann–Whitney *U* test, and Kruskal-Wallis test were used to calculate the differences between the parameters. Bivariate correlations between study variables were calculated using the Spearman’s rank correlation coefficient. Univariate survival analyses were calculated with Cox’s model, and survival curves were plotted with the Kaplan–Meier method. Cox’s model was used also for multivariate survival analyses. In the multivariate analyses, we included the quantity of BATMs, the quantity of BTSMs, patient age, and further variables with a *p* value < 0.05 in univariate analyses.

## Results

### Clinical and pathological characteristics of the BC cohort and quantification of BATMs and BTSMs

The distribution of clinical and pathological parameters in our total cohort and in the BATM/BTSM-high and BATM/BTSM-low subgroup is displayed in Table 1. Not all pathological parameters could be obtained from every patient, which is why the numbers of patients in some subgroups is lower than the number of all cases analyzed. In the overall cohort, 75.8% of all patients were older than 50 years. Most tumors were ER positive (80.9%), PR positive (57.0%), did not show an HER2 amplification (88.3%), and had low proliferation rates (ki-67 ≤ 14%: 56%). 55.7% of all tumors were defined as luminal A-like, 20.1% were luminal B-like, and 12.8% were TNBC. 8.1% of all patients had HER2 amplified luminal-like BC, and only 2.7% were HER2 amplified non-luminal like. Tumor grading was only available in 54.3% of all cases, so this parameter must be regarded with limited reliability (Table 1).

**Table 1:** Distribution of BATMs and BTSMs compared to clinical and pathological parameters in our BC cohort

Parameters	Total	BATM-low	BATM-high	BTSM-low	BTSM-high
	Number of cases (%)	Number of cases (%)	Number of cases (%)	Number of cases (%)	Number of cases (%)
Age					
≥ 50 years	226 (75.8)	123 (75.0)	41 (25.0)	31 (17.2)	149 (82.3)
<50 years	72 (24.2)	47 (83.9)	9 (16.1)	11 (16.7)	51 (77.3)
Chi-square *p* value		0.169		0.926	
Molecular subtype					
Luminal A-like	166 (55.7)	99 (78.0)	28 (22.0)	37 (26.8)	101 (73.2)
Luminal B-like	60 (20.1)	39 (79.6)	10 (20.4)	2 (3.8)	50 (96.2)
Triple negative	38 (12.8)	16 (66.7)	8 (33.3)	0 (0.0)	30 (100.0)
HER2 amplified luminal-like	24 (8.1)	11 (78.6)	3 (21.4)	1 (6.3)	15 (93.7)
HER2 amplified non luminal-like	8 (2.7)	5 (83.3)	1 (16.7)	2 (33.3)	4 (66.7)
Chi square *p* value		0.779		0.000011***	
Grading					
G1	15 (5)	7 (63.6)	4 (36.4)	4 (30.8)	9 (69.2)
G2	103 (34.6)	56 (77.8)	16 (22.2)	12 (15.0)	68 (85.0)
G3	44 (14.8)	25 (78.1)	7 (21.9)	7 (20.6)	27 (79.4)
Chi-square *p* value		0.594		0.337	
Tumor foci					
Unifocal	161 (54.0)	87 (74.4)	30 (25.6)	21 (16.0)	110 (84.0)
Multifocal/multicentric	137 (46.0)	83 (80.6)	20 (19.4)	21 (18.9)	90 (81.1)
Chi-square *p* value		0.272		0.554	
Tumor size					
pT1	194 (65.1)	114 (80.3)	28 (19.7)	31 (20.0)	124 (80.0)
pT2	87 (29.2)	49 (75.4)	16 (24.6)	8 (11.1)	64 (88.9)
pT3	4 (1.3)	1 (33.3)	2 (66.7)	0 (0.0)	3 (100.0)
pT4	13 (4.4)	6 (60.0)	4 (40.0)	3 (25.0)	9 (75.0)
Chi-square *p* value		0.094		0.276	
Axillary lymph node status					
pN0	164 (55.0)	91 (77.8)	26 (22.2)	23 (17.6)	108 (82.4)
pN1	124 (41.6)	73 (76.8)	22 (23.2)	18 (17.6)	84 (82.4)
pN2	4 (1.3)	1 (33.3)	2 (66.7)	0 (0.0)	4 (100.0)
Chi-square *p* value		0.224		0.796	
ER status					
Negative	57 (19.1)	28 (75.7)	9 (24.3)	4 (8.9)	41 (91.1)
Positive	241 (80.9)	142 (77.6)	41 (22.4)	38 (19.3)	159 (80.7)
Chi-square *p* value		0.799		0.096	
PR status					
Negative	128 (43.0)	67 (75.3)	22 (24.7)	20 (25.0)	80 (75.0)
Positive	170 (57.0)	103 (78.6)	28 (21.4)	22 (15.5)	120 (84.5)
Chi-square *p* value		0.561		0.362	
HER2 status					
Negative	263 (88.3)	154 (77.4)	45 (22.6)	39 (17.8)	180 (82.2)
Positive	33 (11.1)	16 (76.2)	5 (23.8)	3 (13.0)	20 (87.0)
Chi-square *p* value		1.000		0.774	
Expression of Ki-67					
≤ 14%	167 (56.0)	100 (78.1)	28 (21.9)	37(26.6)	102 (73.4)
>14%	60 (20.1)	39 (79.6)	10 (20.4)	2(3.8)	50 (96.2)
Chi-square *p* value		0.832		0.001**	
Expression of EP3					
Low (IRS ≤ 1)	87 (29.2)	43 (68.3)	20 (31.7)	16 (22.2)	56 (77.8)
High (IRS >1)	201 (67.4)	124 (80.5)	30 (19.5)	26 (15.5)	142 (84.5)
Chi-square *p* value		0.051		0.208	

A successful staining of BATMs was achieved in 220/298 patient samples (due to technical issues). 22.7% of these cases showed high populations of BATMs while the remaining 77.3% showed a low amount of BATMs (Table 1). BTSMs could be stained in 242/298 samples. High amounts of BTSMs were detected in 82.7% of these cases and a low quantity of BTSMs in 17.3% (Table 1). The quantities of BATMs and BTSMs correlated strongly with each other (*r* = 0.5, *p* = 2.98E−15) (Fig. 1e).

### Association of BATMs and BTSMs with clinical and pathological parameters in BC

The distribution of clinical and pathological parameters in the BATMs/BTSMs high and low subgroups is displayed in Table 1; significant associations of BATMs/BTSMs with clinical and pathological parameters are in Fig. 1.

The subgroups did not correlate to patient age; however, the total quantity of BATMs was significantly higher in patients older than 50 years compared to younger patients (*p* = 0.0325, Fig. 1a, b, f). No further correlations of BATMs and clinical or pathological parameters could be found, neither when analyzing the subgroups, nor when regarding the total quantity of BATMs.

The BTSM subgroup correlated strongly to the molecular subtype (*p* = 0.000011, Table 1). All (100%) TNBC cases belonged to the “BTSM-high” subgroup. The “BTSM-high” subgroup was also strongly represented in luminal B-like tumors—in both HER2-negative and HER2-positive cases. Luminal A-like tumors showed high BTSMs populations less frequently, which was significantly different to the amount in TNBC in a pairwise comparison (*p* = 0.0005, Fig. 1g). The BTSM-high subgroup was least represented in HER2 positive non-luminal cases; however, as this subgroup contained only 6 cases, this result must be regarded with limited reliability. Not only when grouping the quantity of BTSMs into a high and low population, but also when comparing the absolute quantity of BTSMs between the different molecular BC subtypes, a significant association could be found (*p* = 0.0003, Fig. 1g). As the molecular subtypes are defined by surrogate immunohistochemical parameters, these results were consistent when not the subtype but the single parameter was analyzed: high populations of BTSMs occurred more frequently in the ki-67 > 14% group (96.2%) than in cases with an expression of ki-67 ≤ 14% (73.4%, *p* = 0.001) (Table 1). The absolute quantity of BTSMs in ER negative patients was significantly higher than in ER positive patients (*p* = 0.002, Fig. 1h). No correlations between the amount of BTSMs and PR status, HER2 status, or other clinical and pathological parameters were found (Table 1).

### Both BATMs and BTSMs were negatively associated with OS

**Fig. 2:**
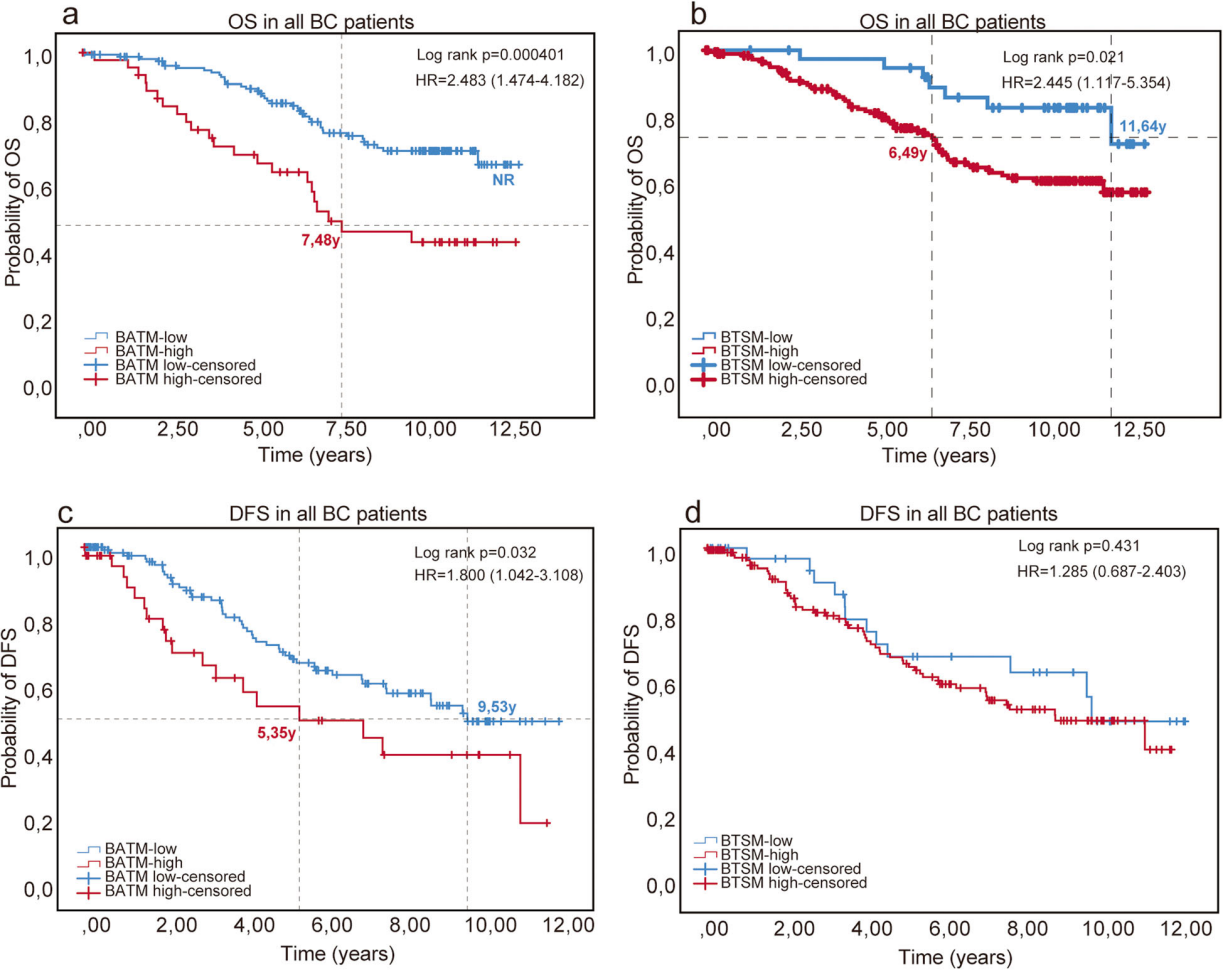
BATMs and BTSMs and clinical outcome in BC. The quantity of BATMs significantly correlated to OS and DFS of BC patients: Median OS was 7.48 years in the BATM-high subgroup compared to not reached (NR) in the BATM-low subgroup (HR = 2.483, *p* = 0.000401, **a**). Median DFS was 5.35 years in the BATM-high subgroup compared to 9.53 years in the BATM-low subgroup (HR = 1.800, *p* = 0.032, **c**). The quantity of BTSMs significantly correlated to OS but not to DFS of BC patients: 75% OS was 6.49 years in the BTSM-high subgroup compared to 11.64 years in the BTSM-low subgroup (HR = 2.445, *p* = 0.021, **b**). There was no significant association of BTSM with DFS (HR = 1.285, *p* = 0.431, **d**). BTAMs, breast adipose tissue macrophages; BTSMs, breast tumor-stroma macrophages; OS, overall survival; DFS, disease-free survival; HR, hazard ratio

Patients in the BATM-high (HR = 2.483, *p* = 0.000401, Fig. 2a) as well as in the BTSM-high (HR = 2.445, *p* = 0.021, Fig. 2b) subgroup showed a significantly impaired OS compared to the respective “low” subgroup. Median OS was 7.48 years in the BATM-high population (*n* = 50) while the median was not reached (NR) in our follow-up period in the BATM-low (*n* = 170) population. Seventy-five percent OS was 6.49 years in the BTSM-high (*n* = 200) versus 11.64 years in BTSM-low (*n* = 42) subgroup. Median OS was not reached in the BTSM-high as well as in the BTSM-low subgroup.

In a subgroup analysis, a high amount of BATMs was a negatively associated with OS independent of tumor foci (in unifocal tumors: Additional file 1a, *n* = 117, *p* = 0.006 and in multifocal and multicentric tumors: additional file 1b, *n* = 103, *p* = 0.025) and independent of PR status (in PR positive tumors: Additional file 1d, *n* = 131, *p* = 0.041 and in PR-negative tumors, Additional file 1e, *n* = 89, *p* = 0.005). High amounts of BATMs were also negatively associated with OS in the subgroups of patients aged older than 50 years (Additional file 1c, *n* = 164, *p* = 0.001), in luminal A-like tumors (Additional file 1f, *n* = 127, *p* = 0.001), in TNBC (Additional file 1 g, *n* = 24, *p* = 0.049), in tumors smaller than 2 cm in size (Additional file 1 h, *n* = 142, *p* = 0.008), in BC patients with negative lymph node status (Additional file 1i, *n* = 117, *p* = 0.00021), in ER-positive tumors (Additional file 1j, *n* = 183, *p* = 0.003), in HER2-negative tumors (Additional file 1 k, *n* = 199, *p* = 0.001), and in tumors with a low proliferation rate (Ki-67 ≤ 14%) (Additional file 1l, *n* = 128, *p* = 0.001). No significant correlations between the amount of BATMs and OS were seen in in the respective other subgroups.

In the subgroup analysis, a high amount of BTSMs was a negatively associated with OS in the subgroup of patients aged older than 50 years (Additional file 2a, *n* = 180, *p* = 0.029), in luminal A-like tumors (Additional file 2b, *n* = 138, *p* = 0.046), in multifocal and multicentric tumors (Additional file 2c, *n* = 111, *p* = 0.026), in tumors smaller than 2 cm in size (Additional file 2d, *n* = 155, *p* = 0.031), in BC patients with negative lymph node status (Additional file 2e, *n* = 131, *p* = 0.029), in ER-positive tumors (Additional file 2f, *n* = 197, *p* = 0.034), in HER2-negative tumors (Additional file 2 g, *n* = 219, *p* = 0.026), and in tumors with a low proliferation rate (Ki67 ≤ 14%) (Additional file 2h, *n* = 139, *p* = 0.039). No significant correlations between the amount of BTSMs and OS were seen in the respective other subgroups.

### Only BATMs but not BTSMs were negatively associated with DFS

In the overall patient cohort, the BATM-high subgroup showed a significantly impaired DFS compared to patients with low amounts of BATMs (median DFS 5.35 years in the BATM-high (*n* = 50) vs. 9.53 years in the BATM-low (*n* = 170) population, HR = 1.800, *p* = 0.032, Fig. 2c). There was no significant association of the amount of BTSMs with DFS, neither in the overall cohort (HR = 1.285 *p* = 0.431, Fig. 2d), nor in a subgroup analysis.

A subgroup analysis revealed that BATMS were negatively associated with DFS in the subgroups of patients aged older than 50 years (Additional file 1 m, *n* = 164, *p* = 0.015) and in patients with unifocal tumors (Additional file 1n, *n* = 117, *p* = 0.016). No significant correlations between the amount of BATMs and DFS were seen in the respective other subgroups.

### BATMS were independently associated with OS in BC

Univariate COX regression analysis showed that the BATMs subgroup (*p* = 0.001, HR = 2.483, 95% CI 1.474–4.182), the BTSMs subgroup (*p* = 0.025, HR = 2.445, 95% CI 1.117–5.354), the molecular subtype (*p* = 0.03, HR = 1.213, 95% CI 1.081–1.444), grading (*p* = 0.003, HR = 1.763, 95% CI 1.056–2.945), tumor size (*p* = 5.57E−13, HR = 2.064, 95% CI 1.695–2.513), axillary lymph node status (*p* = 0.002, HR = 1.859, 95% CI 1.256–2.749), and ER status (*p* = 0.026, HR = 0.589, 95% CI 0.369–0.940) were significantly associated with OS (Table 2).

**Table 2:** Univariate and multivariate Cox regression analyses of OS including various prognostic parameters in patients with BC

	Univariate analysis			Multivariate analysis model 3 (with both BATM and BTSM)		
	*p*	HR	95% CI	*p*	HR	95% CI
Age (<50 years vs. ≥ 50 years)	0.055	1.746	0.987–3.088	0.741	0.843	0.305–2.326
Molecular subtype (LuA-like vs. LuB-like vs. TNBC vs. HER2 Lu-like vs. HER2 nonLu like)	0.03*	1.213	1.018–1.444	0.185	1.351	0.866–2.107
Grading (G1 vs. G2 vs. G3)	0.003*	1.763	1.056–2.945	0.405	1.403	0.633–3.110
Tumor foci (unifocal vs. multifocal and multicentric)	0.889	0.971	0.642–1.469	n.i.	n.i.	n.i.
Tumor size (pT1 vs. pT2 vs. pT3 vs. pT4)	5.573E−13*	2.064	1.695–2.513	0.001*	1.827	1.269–2.631
Axillary lymph node status (pN0 vs. pN1 vs.pN2)	0.002*	1.859	1.256–2.749	0.285	1.650	0.659–4.131
ER status (ER− vs. ER+)	0.026*	0.589	0.369–0.940	0.604	0.718	0.205–2.515
PR status (PR− vs. PR+)	0.088	0.697	0.461–1.054	n.i.	n.i.	n.i.
HER2 status (HER2− vs. HER2+)	0.079	1.667	0.942–2.952	n.i.	n.i.	n.i.
Expression of ki-67 (ki-67 ≤ 14% vs. ki-67>14%)	0.891	1.040	0.588–1.840	n.i.	n.i.	n.i.
BATMs (low vs. high)	0.001*	2.483	1.474–4.182	0.004*	4.464	1.624–12.269
BTSMs (low vs. high)	0.025*	2.445	1.117–5.354	0.737	0.813	0.243–2.721

Multivariate analysis model 3 was performed with both BATMs and BTSMs, to determine if BATMs, BTSM or both were independently associated with OS when both subtypes of macrophages were considered. *ER* estrogen receptor, *PR* progesterone receptor, *HER2* human epidermal growth factor receptor 2, *LuA-like* luminal A-like, *LuB-like* luminal B-like, *TNBC* triple-negative breast cancer, *HER2 Lu-like* HER2 amplified luminal-like, *HER2 nonLu like* HER2 amplified non luminal-like, *BATMs* breast adipose tissue macrophages, *BTSMs* breast tumor-stroma macrophages, *HR* hazard ratio, *CI* confidence interval, *n.i* not included in multivariate model as *p* > 0.05 in univariate analysis; *significant (*p* value < 0.05)

Multivariate analysis was performed in three different models. All models included the univariate significant parameters age, molecular subtype, grading, tumor size, and axillary lymph node status. Multivariate analysis model 1 was performed including BTSMs but without BATMs, to determine if BTSMs were independently associated with OS in the whole patient cohort. Multivariate model 2 was performed including BATMs but without BTSMs, to determine if BATMs were independently associated with OS in the whole patient cohort. Multivariate analysis model 3 was performed with both BATMs and BTSMs, to determine if BATMs, BTSMs, or both were independently associated with OS when both subtypes of macrophages were considered. As shown in Additional file 3, the multivariate COX regression analysis model 1 revealed that tumor size (*p* = 0.001, HR = 1.873, 95% CI 1.304–2.689) was independently associated with OS, but BTSMs were not. The multivariate COX regression analysis model 2 revealed that BATMs (*p* = 0.002, HR = 4.259, 95% CI 1.666–10.887) and tumor size (*p* = 0.001, HR = 1.847, 95% CI 3.113–1093.217) were independently associated with OS. The multivariate COX regression analysis model 3 also showed that BATMs (*p* = 0.004, HR = 4.464, 95% CI 1.624–12.269) and tumor size (*p* = 0.001, HR = 1.827, 95% CI 1.269–2.631) were independently associated with OS. So, even when BTSMs were taken into consideration, BATMs remained associated with OS of BC patients (Table 2).

### BATMs were independently associated with DFS in BC

Univariate COX regression analysis showed that the BATMs subgroup (*p* = 0.035, HR = 1.800, 95% CI 1.042–3.108), tumor grade (*p* = 0.03, HR = 1.669, 95% CI 1.050–2.654), tumor size (*p* = 0.002, HR = 1.493, 95% CI 1.159–1.922), and axillary lymph node status (*p* = 0.01, HR = 1.696, 95% CI 1.137–2.528) were significantly associated with DFS a in our BC cohort (Table 3). BTSMs were not associated with DFS in the univariate analysis.

**Table 3:** Univariate and multivariate Cox regression analyses of DFS including various prognostic parameters in patients with BC

	Univariate analysis			Multivariate analysis		
	*p*	HR	95% CI	*p*	HR	95% CI
Age(<50 years vs. ≥ 50 years)	0.19	0.732	0.459–1.167	0.134	0.537	0.238–1.210
Molecular subtype (LuA-like vs. LuB-like vs. TNBC vs. HER2 Lu-like vs. HER2 nonLu like)	0.329	1.093	0.914–1.307	n.i.	n.i.	n.i.
Grading (G1 vs. G2 vs. G3)	0.03*	1.669	1.050–2.654	0.043*	1.825	1.018–3.271
Tumor foci (unifocal vs. multifocal and multicentric)	0.370	1.214	0.794–1.857	n.i.	n.i.	n.i.
Tumor size (pT1 vs. pT2 vs. pT3 vs. pT4)	0.002*	1.493	1.159–1.922	0.011*	1.646	1.120–2.418
Axillary lymph node status (pN0 vs. pN1 vs.pN2)	0.01*	1.696	1.137–2.528	0.856	1.078	0.482–2.411
ER status (ER− vs. ER+)	0.771	0.926	0.550–1.557	n.i.	n.i.	n.i.
PR status (PR− vs. PR+)	0.249	1.291	0.836–1.994	n.i.	n.i.	n.i.
HER2 status (HER2− vs. HER2+)	0.511	1.228	0.666–2.262	n.i.	n.i.	n.i.
Expression of ki-67 (ki-67 ≤ 14% vs. ki-67>14%)	0.093	1.569	0.928–2.653	n.i.	n.i.	n.i.
BATMs (low vs. high)	0.035*	1.800	1.042–3.108	0.005*	3.240	1.423–7.378
BTSMs (low vs. high)	0.432	1.285	0.687–2.403	n.i.	n.i.	n.i.

*ER* estrogen receptor, *PR* progesterone receptor, *HER2* human epidermal growth factor receptor 2, *LuA-like* luminal A-like, *LuB-like* luminal B-like, *TNBC* triple-negative breast cancer, *HER2 Lu-like* HER2 amplified luminal-like, *HER2 nonLu like* HER2 amplified non luminal-like, *BTAMs* breast adipose tissue macrophages, *BTSMs* breast tumor-stroma macrophages, *HR* hazard ratio, *CI* confidence interval, *n.i* not included in multivariate model, as *p* > 0.05 in univariate analysis, *significant (*p* value < 0.05)

The multivariate analysis included the parameters age, grading, tumor size, axillary lymph node status, and the BATM subgroup. As shown in Table 3, the multivariate COX regression analysis revealed that the BATM subgroup (*p* = 0.005, HR = 3.240, 95% CI 1.423–7.378), grading (*p* = 0.043, HR = 1.825, 95% CI 1.018–3.271), and tumor size (*p* = 0.011, HR = 1.646, 95% CI 1.120–2.418) were independently associated with DFS in patients with BC (Table 3).

### BATMs correlated negatively to EP3 expression and a combination of both parameters identified a subgroup with extremely poor OS and DFS

**Fig. 3:**
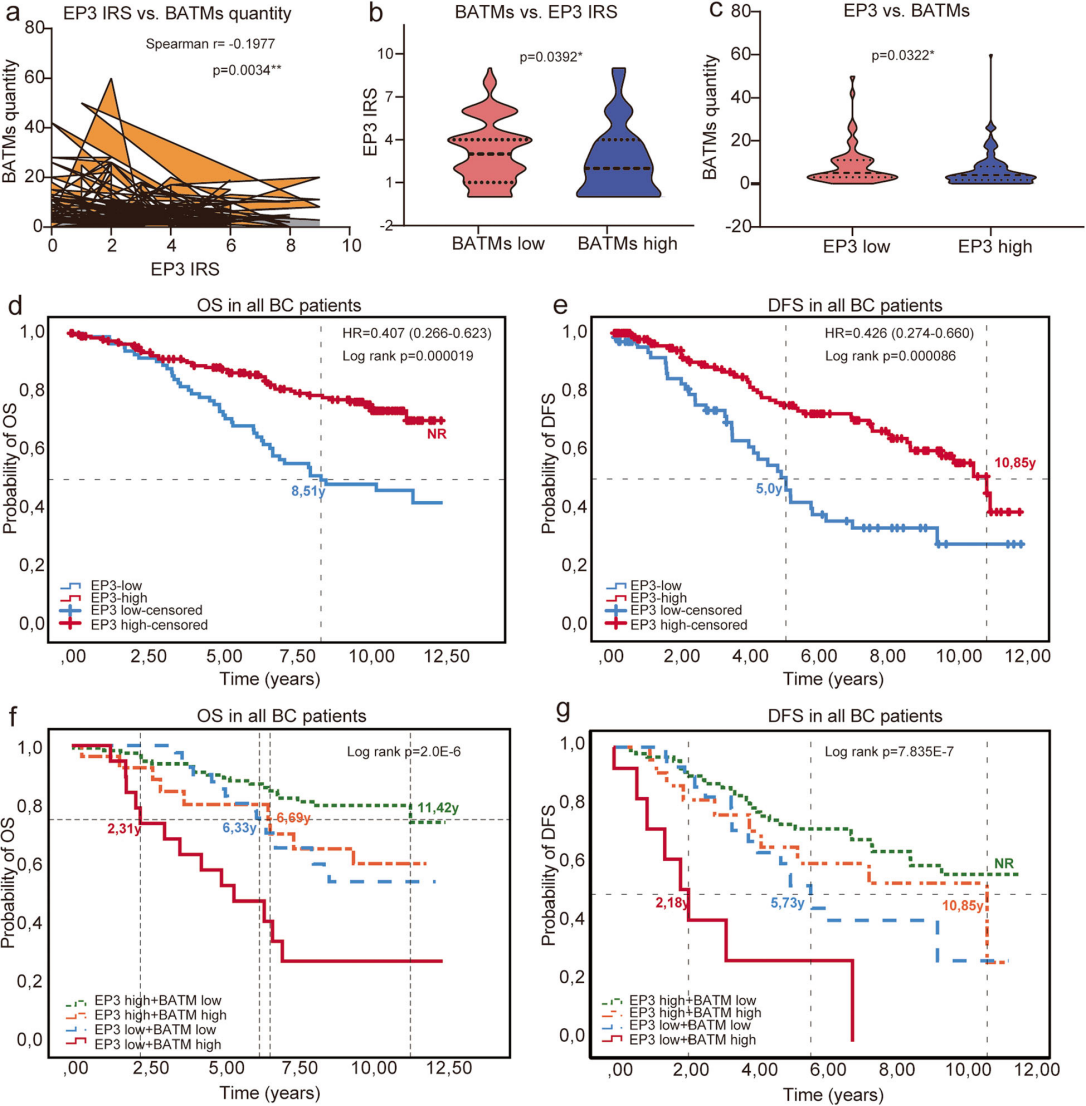
The quantity of BATMs was negatively associated with EP3 expression in BC and the combination of both parameters significantly correlated to OS and DFS. There was a negative correlation between the quantity of BATMs and the EP3 immunoreactive score (IRS) (**a**). Tumors in the BTAMs high subgroup showed lower EP3 expression than tumors in the BTAMs low subgroup (**b**). In the EP3-low subgroup, higher quantities of BATMs occurred than in the EP3-high subgroup (**c**). OS and DFS were superior in patients with high expression of EP3 compared to EP3 low expressing BC patients (**d**, **e**). Patients in the combined subgroup “EP3-low/BATM-high” had the highest mortality risk and highest recurrence risk among all combinations of EP3 high/low and BATMs high/low in the survival analysis (**f**, **g**). BATMs, breast adipose tissue macrophage; EP3, prostaglandin E receptor 3; OS, overall survival; DFS, disease-free survival

The quantity of BATMs (continuous variable) correlated negatively to the EP3 expression quantified by the IRS (continuous variable, spearman *r* = − 0.1977, *p* = 0.0034, Fig. 3a). In the BATM-high subgroup, the EP3 expression was significantly lower than in the BATM-low subgroup (*p* = 0.00392) (Fig. 3b). Similarly, when categorizing EP3 in an EP3-high (IRS > 1) and an EP3-low (IRS ≤ 1) expressing subgroup, there were higher quantities of BATMs in the EP3-low than in the EP3-high subgroup. (*p* = 0.0322, Fig. 3c). Comparing both categorized variables (BATM-high and -low with EP3-high and -low), a correlation of only borderline significance could be found (*p* = 0.051, Table 1). No correlations between EP3 expression and the quantity of BTSMs could be shown.

Consistent with the previous study by our team (in which patients with DCIS were not excluded) [24], OS and DFS were superior in invasive BC patients with high expression of EP3 compared to patients with low EP3 expression (OS: *p* = 0.000019, HR = 0.407, 95% CI 0.266–0.623), Fig. 3d and DFS: *p* = 0.000086, HR = 0.426, 95% CI 0.274–0.660, Fig. 3e). We defined four subgroups using combinations of the categorized variables EP3-high/-low and BATM-high/low. Doing so, we could identify a subgroup with an extremely poor prognosis: patients in the subgroup “EP3-low/BATM-high” (*n* = 20) showed the worst survival rates—median OS 2.31 years and median DFS 2.18 years—compared to the group “EP3-high/BATM-low” (*n* = 124) with the best prognosis (OS: median OS 11.42 years, *p* = 0.000002, Fig. 3f and Table 4, part A; DFS: median DFS NR, *p* = 0.000005, Fig. 3g and Table 4, part A). In other words, patients in the “EP3-low/BATM-high” subgroup had a 1.756 times higher mortality risk and 1.922 times higher recurrence risk than patients in the favorable “EP3-high/BATM-low” subgroup (Table 4 part A). Moreover, the association of BATMs with OS was different considering EP3 expression: in the BATM-high subgroup, EP3-low-expressing patients had a 1.647 times higher mortality risk (*p* = 0.023) and 2.107 times higher recurrence risk (*p* = 0.004) than EP3-high-expressing patients (Table 4, part B). But EP3 was also associated with survival in the BATM-low subgroup, where EP3-low-expressing patients had a 1.511 times higher mortality risk (*p* = 0.01) and 1.405 times higher recurrence risk (*p* = 0.028) than EP3-high-expressing patients (Table 4, part C). Interestingly, BATMs on the other hand did not modify the positive prognostic association of a high EP3-expression: in the EP3-high subgroup, OS and DFS were not significantly different between BATM-low and BATM-high patients (OS: *p* = 0.059; DFS: *p* = 0.326, Table 4 part D). Only in the EP3-low subgroup, BATMs were significantly correlated to OS and the BATM-high subgroup had a 2.722 times higher mortality risk (*p* = 0.007) and a 4.049 times higher recurrence risk (*p* = 0.002) than the BATM-low subgroup (Table 4, part E).

**Table 4:** BC survival analysis using a combination of the prognostic factors EP3 and BATMs

	OS%	*p* value	HR	95% CI	DFS%	*p* value	HR	95% CI
A.								
EP3 high+BATM low	83.1				77.4			
EP3 high+BATM high	70.0	0.059	2.128	0.973–4.658	66.7	0.326	1.437	0.697–2.962
EP3 low+BATM low	58.1	0.01*	1.511	1.102–2.070	60.5	0.028*	1.405	1.037–1.902
EP3 low+BATM high	35.0	0.000002*	1.756	1.391–2.217	60.0	0.000005*	1.922	1.453–2.544
B.								
BATM high+EP3 high	70.0				66.7			
BATM high+EP3 low	35.0	0.023*	1.647	1.070–2.536	60.0	0.004*	2.107	1.264–3.513
C.								
BATM low+EP3 high	83.1				77.4			
BATM low+EP3 low	58.1	0.01*	1.511	1.102–2.070	60.5	0.028*	1.405	1.037–1.902
D.								
EP3 high+BATM low	83.1				77.4			
EP3 high+BATM high	70.0	0.059	2.128	0.973–4.658	66.7	0.326	1.437	0.697–2.962
E.								
EP3 low+BATM low	58.1				60.5			
EP3 low+BATM high	35.0	0.007*	2.722	1.321–5.609	60.0	0.002*	4.049	1.665–9.847

*BATMs* breast adipose tissue macrophage, *EP3* prostaglandin E receptor 3, *OS* overall survival, *DFS* disease-free survival, *p p* value, *HR* hazard ratio, *CI* confidence interval; *significant (*p* value < 0.05)

## Discussion

In this study of 298 sporadic invasive BC cases, we could demonstrate that the abundance of macrophages not only in the tumor-stroma (BTSMs) but also in the breast adipose tissue (BATMs) was negatively associated with OS. We could also show that BATMs were negatively associated with DFS. Furthermore, BATMs were an independent prognostic parameter for both OS and DFS in BC in a multivariate analysis while BTSMs were not. This suggests that local breast adipose tissue might be associated with BC outcome.

The relationship between adipocytes in adipose tissue of obese BC patients and macrophages has been well documented in recent years [34, 45]. However, our study did not focus on adipose tissue due to obesity nor did we include obese patients only—it aimed to analyze the role of the adipose tissue right in the breast that is also present in lean BC patients. We therefore observed for the first time in the current study that local adipose tissue might have a negative association with BC survival, if elevated rates of macrophages occur in it. BATMs might initiate or enhance tumorigenic immune effects in BC.

However, up to now we, could not reveal where the BATMs originate from. Possible theories are as follows: (i) BATMs are derived from BTSMs that migrate to adipose tissue by chemotaxis, (ii) BATMs are derived from monocytes that migrate into the breast adipose tissue due to adipocyte-derived adipokines and differentiate into a specific adipose-tissue associated macrophage phenotype, or (iii) BATMs are derived from both sources named above.

In our study, we found a strong correlation between the two types of macrophages (BATMs vs. BTSMs), which might lead to the conclusion that BTSMs are the source of BATMs. However, although the quantity of BATMs and BTSMs correlated strongly, their associations with clinical parameters and outcome were not the same. This favors the theory of an origin of the BATMs from the blood monocyte/macrophage cell lineage. Most probably, BATMs are not derived from a single but from both sources. In the condition of obesity, the continuous flow and accumulation of macrophages from the blood circulation to the adipose tissue is an important process that initiates the chronic inflammation in the adipose tissue of obese individuals [46]. However, without the condition of obesity, it is still not known if a similar adipose tissue-macrophage crosstalk exists. Further research might help to answer this question.

We further found that EP3 expression was negatively correlated to BATM quantity and that the association of BATMs with survival outcomes was different considering EP3 expression. EP3 has shown a positive prognostic association with BC survival in our previous study [24], similar to EP1 [25], but contrary to the negative effects of EP2 and EP4 [21, 23]. However, the positive prognostic role of EP3 could not be explained by tumor cell biology, which resulted in the hypothesis that EP3-mediated effects in BC might be modulated by other aspects, like immunological factors in the tumor environment [47]. As the present study now shows how EP3 is correlated to BATMs, EP3 might be involved in the regulation of the occurrence or the phenotype of BATMs in BC, which might explain the observed positive association of EP3 with BC survival. Intriguingly, a recent study pointed out that EP3 could induce an interleukin-13 (IL-13)-mediated polarization of macrophages from a pro-inflammatory to a pro-reparative phenotype during liver repair [48]. Similar mechanisms of EP3 influencing macrophage polarization, e.g., reducing the pro-tumorigenic effect of BATMs by converting or alternating the polarized phenotype, might exist in BC. Our further studies aim to clarify how EP3 might regulate BATMs in BC.

In contrast to BATMs, the amount of BTSMs was particularly associated with the BC molecular subtype in our study. In TNBC, BTSMs were highest among all five molecular subtypes. Similarly, BTSMs were associated with a negative ER status and ki-67 rates > 14%. In conclusion, high amounts of BTSMs were associated with aggressive clinical features. Especially in TNBC, options for targeted therapies are in the focus of recent research to improve the prognosis of this patient subgroup [49]. BTSMs might offer an option for a future targeted therapy and might additionally serve as prognostic factor to stratify patients’ risk and to choose the appropriate therapy [27, 50, 51].

We found a significant association of BATMs and BTSMs with the prognosis of BC patients not only in the overall cohort, but also in different subgroups. This indicates that the prognostic role of macrophages can differ dependently on different tumor context. This supports the thesis that the development of BC is not only controlled by a single molecule abnormality, but also by the interplay between BC cells and the whole tumor microenvironment (TME) [52]. Besides the number of tumor-associated macrophages, their polarization is also relevant for tumor development [53–55]. As research on the polarization state of macrophages has become more abundant and in-depth, recent studies have shown that their occurrence in tumors and their influence on tumor development is more complicated than initially thought. Macrophages in the tumor microenvironment are not limited to the M1 or M2 phenotype, but can reside in between or outside the spectrum [56]. In white adipose tissue during obesity, a complex mixture of M1 and M2 macrophages phenotypes can be observed [57]. This indicates that also BATMs cannot be classified using the simple dual M1/M2 model. The removal of all macrophage populations, regardless of their polarization status, has remarkable influence: The occurrence of primary and metastatic tumors was significantly reduced due to macrophage depletion [56]. However, that study did not distinguish between BTSMs and BATMs. So, in the context of BC immunotherapy, enhancing the understanding of the specific roles of BATMS and BTSMs, respectively, might seem to be as or even more important than characterizing their phenotype as M1 or M2. To deeply understand how BATMs might be involved in BC development and to evaluate prevention and treatment strategies thoroughly, a clear analyzation of the BATMs subgroup is crucial.

## Conclusion

An abundance of BATMs in BC was an independent and highly significant prognostic factor for an impaired OS and DFS. The quantity of BATMs correlated significantly with the amount of BTSMs; however, BTSMs were not an independent prognostic factor for neither OS nor DFS. The amount of BTSMs, in contrast, correlated significantly with the molecular subtype and was especially high in TNBC. Therefore, it is essential to keep in mind that research on the role of macrophages in BC should not just focus on M1 or M2 polarization, but also on the exact localization of macrophages in the TME. We could demonstrate that the subpopulations of BTSMs and BATMs might affect the overall development of BC together but each subpopulation in a different way. As their quantity is significantly related to each other, both subgroups seem to depend on each other. Most importantly, our findings suggest that breast adipose tissue might contribute to the aggressiveness of BC via BATMs. Targeting BATMs might be a promising strategy in future BC therapies.

## Supplementary Information

**Additional file 1 **BATMs significantly correlated to both OS and DFS in some clinical subpopulations of BC patients. BATMs were a negative prognostic factor for OS independent of tumor foci (unifocal, *p* = 0.006 and multifocal and multicentric tumors, *p* = 0.025) **(a-b)** and independent of PR status (PR positive tumors, *p* = 0.041 and PR negative tumors, *p* = 0.005) **(d-e)**. It further showed prognostic impact on OS in the subgroups of patients aged older than 50 years (*p* = 0.001, **c**), in Luminal A-like tumors (*p* = 0.001, **f**), in TNBC tumors (*p* = 0.049, **g**), in tumors smaller than 2 cm in size (*p* = 0.008, **h**), in BC patients with negative lymph node status (*p* = 0.00021, **i**), in ER positive tumors (*p* = 0.003, **j**), in HER2 negative tumors (*p* = 0.001, **k**) and in tumors with low proliferation rate (ki-67 ≤ 14%) (*p* = 0.001, **l**). BATMs showed prognostic influence on DFS in the subgroups of patients aged older than 50 years (*p* = 0.015, **m**) and in unifocal tumors (*p* = 0.016, **n**). BATMs, Breast adipose tissue macrophages; OS, Overall survival; DFS, Disease-free survival; ER, Estrogen receptor; PR, Progesterone receptor; HER2, Human epidermal growth factor receptor 2.

**Additional file 2 **BTSMs significantly correlated to OS in some clinical subpopulations of BC patients. BTSMs were a negative prognostic factor for OS in the subgroup of patients aged older than 50 years (*p* = 0.029, **a**), in Luminal A-like tumors (*p* = 0.046, **b**), in multifocal and multicentric tumors (*p* = 0.026, **c**), in tumors smaller than 2 cm in size (*p* = 0.031, **d**), in BC patients with negative lymph node status (*p* = 0.029, **e**), in ER positive tumors (*p* = 0.034, f), in HER2 negative tumors (*p* = 0.026, **g**) and in tumors with low proliferation rate (Ki67 ≤ 14%) (*p* = 0.039, **h**). BTSMs, Breast tumor-stroma macrophages; OS, Overall survival; DFS, Disease-free survival; ER, Estrogen receptor; PR, Progesterone receptor; HER2, Human epidermal growth factor receptor 2.

**Additional file 3.** Multivariate Cox regression analyses of OS of various prognostic parameters in patients with BC.

## Data Availability

The datasets generated and/or analyzed during the current study are available from the corresponding author on reasonable request.

## Abbreviations

BATMs: Breast adipose tissue macrophages
BC: Breast cancer
BMI: Body mass index
BTSMs: Breast tumor-stroma macrophages
CI: Confidence interval
CLS: Crown-like structures
DCIS: Ductal carcinoma in situ
DFS: Disease-free survival
EP1: Prostaglandin E receptor 1
EP2: Prostaglandin E receptor 2
EP3: Prostaglandin E receptor 3
EP4: Prostaglandin E receptor 4
ER: Estrogen receptor
HER2: Human epidermal growth factor receptor 2
HER2 Lu-like: HER2 amplified luminal-like
HER2 nonLu like: HER2 amplified non luminal-like
HR: Hazard ratio
HRs: Hormone receptors
IL-4: Interleukin-4
IL-6: Interleukin-6
IL-12: Interleukin-12
IL-13: Interleukin-13
IRS: Immune reactive score
LPS: Lipopolysaccharide
LuA-like: Luminal A-like
LuB-like: Luminal B-like
MCSF: Macrophage colony-stimulating factor
n.i.: Not included
NR: Not reached
OS: Overall survival
PGE2: Prostaglandin E2
PR: Progesterone receptor
RFS: Relapse-free survival
ROC-curve: Receiver operating characteristic curve
TAMs: Tumor-associated macrophages
TME: Tumor microenvironment
TNBC: Triple-negative breast cancer
VLD: Very low density
