# Supplementary figures and images for: Breast adipose tissue macrophages (BATMs) have a stronger correlation with breast cancer survival than breast tumor stroma macrophages (BTSMs)
Source: Breast Cancer Res. 2021 Apr 13;23:45. doi: 10.1186/s13058-021-01422-x (PMC8042723; doi:10.1186/s13058-021-01422-x)

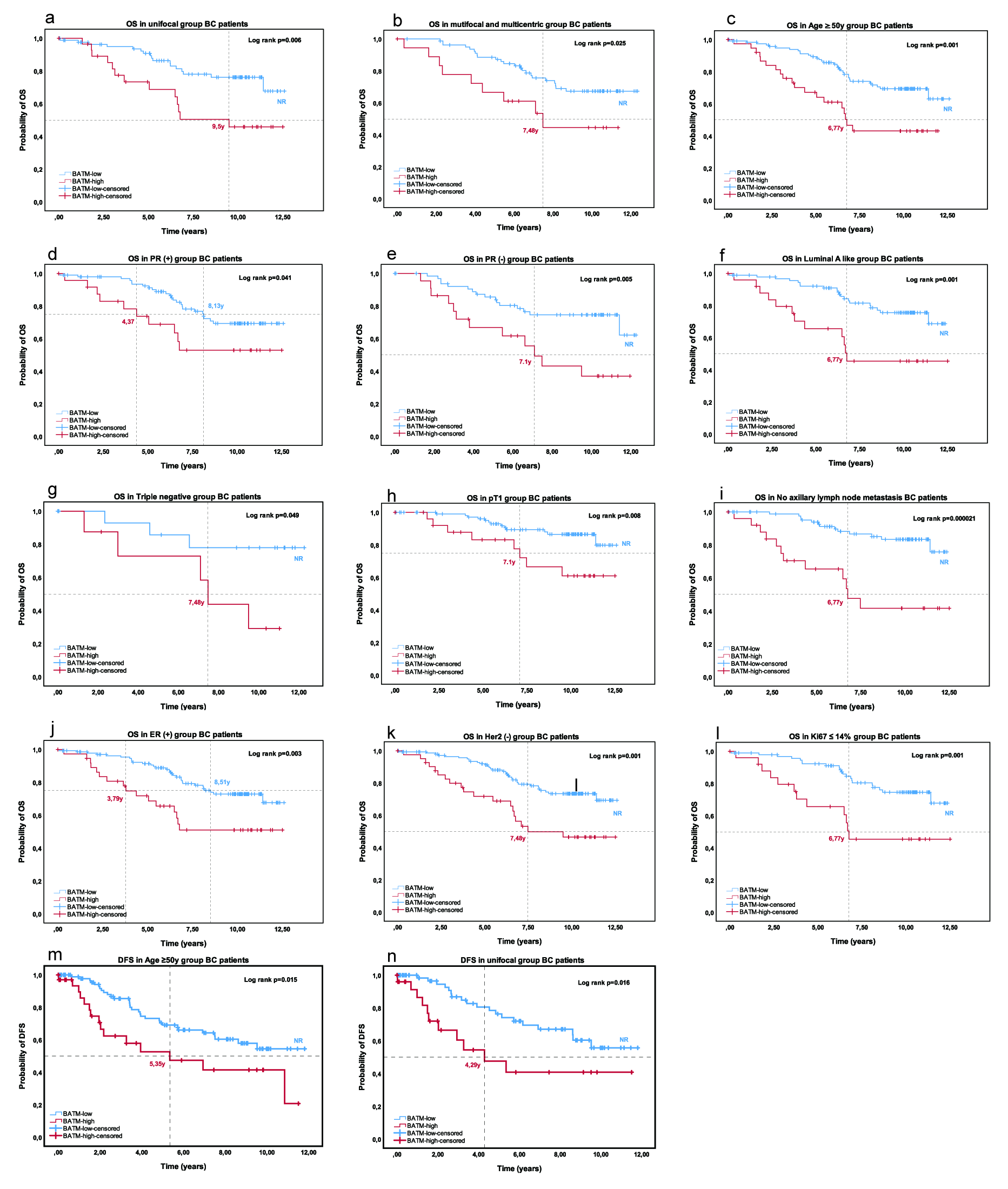

Supplement: Supplementary file 1 — Additional file 1 BATMs significantly correlated to both OS and DFS in some clinical subpopulations of BC patients. BATMs were a negative prognostic factor for OS independent of tumor foci (unifocal, p = 0.006 and multifocal and multicentric tumors, p = 0.025) (a-b) and independent of PR status (PR positive tumors, p = 0.041 and PR negative tumors, p = 0.005) (d-e). It further showed prognostic impact on OS in the subgroups of patients aged older than 50 years (p = 0.001, c), in Luminal A-like tumors (p = 0.001, f), in TNBC tumors (p = 0.049, g), in tumors smaller than 2 cm in size (p = 0.008, h), in BC patients with negative lymph node status (p = 0.00021, i), in ER positive tumors (p = 0.003, j), in HER2 negative tumors (p = 0.001, k) and in tumors with low proliferation rate (ki-67 ≤ 14%) (p = 0.001, l). BATMs showed prognostic influence on DFS in the subgroups of patients aged older than 50 years (p = 0.015, m) and in unifocal tumors (p = 0.016, n). BATMs, Breast adipose tissue macrophages; OS, Overall survival; DFS, Disease-free survival; ER, Estrogen receptor; PR, Progesterone receptor; HER2, Human epidermal growth factor receptor 2. [file 13058_2021_1422_MOESM1_ESM.tif]

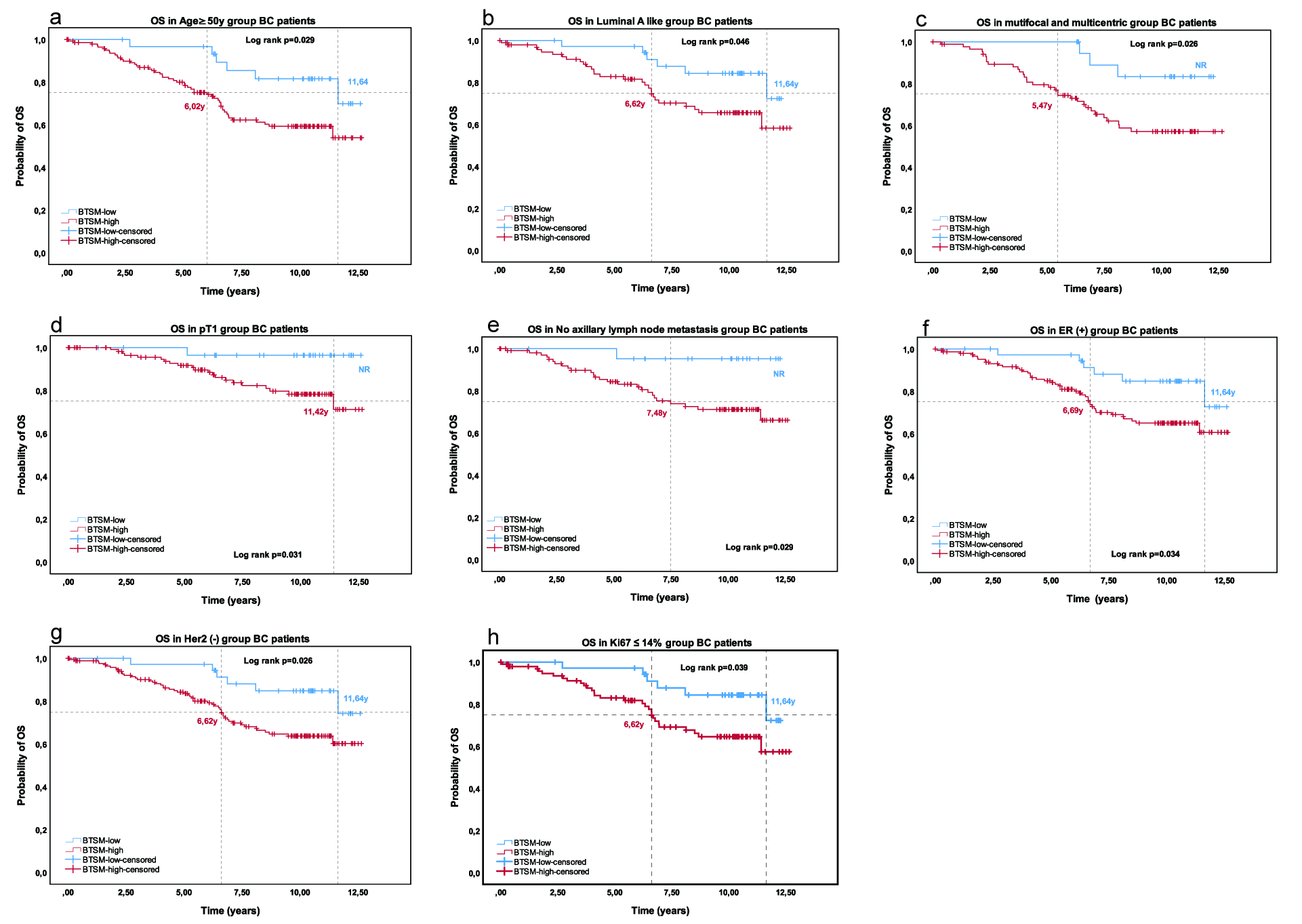

Supplement: Supplementary file 2 — Additional file 2 BTSMs significantly correlated to OS in some clinical subpopulations of BC patients. BTSMs were a negative prognostic factor for OS in the subgroup of patients aged older than 50 years (p = 0.029, a), in Luminal A-like tumors (p = 0.046, b), in multifocal and multicentric tumors (p = 0.026, c), in tumors smaller than 2 cm in size (p = 0.031, d), in BC patients with negative lymph node status (p = 0.029, e), in ER positive tumors (p = 0.034, f), in HER2 negative tumors (p = 0.026, g) and in tumors with low proliferation rate (Ki67 ≤ 14%) (p = 0.039, h). BTSMs, Breast tumor-stroma macrophages; OS, Overall survival; DFS, Disease-free survival; ER, Estrogen receptor; PR, Progesterone receptor; HER2, Human epidermal growth factor receptor 2. [file 13058_2021_1422_MOESM2_ESM.tif]
