# Supplementary material for: Breast adipose tissue macrophages (BATMs) have a stronger correlation with breast cancer survival than breast tumor stroma macrophages (BTSMs)
Source: Breast Cancer Res. 2021 Apr 13;23:45. doi: 10.1186/s13058-021-01422-x (PMC8042723; doi:10.1186/s13058-021-01422-x)
Supplement: Supplementary file 3 — Additional file 3. Multivariate Cox regression analyses of OS of various prognostic parameters in patients with BC. [file 13058_2021_1422_MOESM3_ESM.pdf]

Supplementary table 1. Multivariate Cox regression analyses of OS of various prognostic parameters in patients with BC

	Multivariate analysis model 1 (without BATM)			Multivariate analysis model 2 (without BTSM)		
	p	HR	95% CI	p	HR	95% CI
Age(< 50y vs. ≥50y)	0.874	1.079	0.420-2.773	0.674	0.807	0.298-2.189
Molecular subtype (LuA-like vs.LuB-like vs. TNBC vs. HER2 Lu-like vs. HER2 nonLu-like)	0.332	1.224	0.814-1.841	0.186	1.351	0.865-2.109
Grading (G1 vs. G2 vs. G3)	0.580	1.219	0.604-2.459	0.376	1.434	0.646
Tumor size (pT1 vs. pT2 vs. pT3 vs. pT4)	0.001*	1.873	1.304-2.689	0.001*	1.847	1.283-2.658
Axillary lymph node status (pN0 vs. pN1 vs.pN2)	0.476	1.368	0.578-3.238	0.295	1.620	0.656-4.002
ER status (ER- vs. ER+)	0.775	0.853	0.285-2.548	0.670	0.765	0.224-2.620
BATMs (low vs. high)				0.002*	4.259	1.666-10.887
BTSMs (low vs. high)	0.424	1.529	0.541-4.324			

Multivariate analysis model 1 was performed without BATM, which was attempt to show whether BTSM is an independent prognostic factor of OS of whole patient cohort; multivariate model 2 was performed without BTSM, which was attempt to show whether BATM is an independent prognostic factor of OS of whole patient cohort. ER, Estrogen receptor; HER2, Human Epidermal growth factor receptor 2; LuA-like, Luminal A-like; LuB-like, Luminal B-like; TNBC, Triple negative breast cancer, HER2 Lu-like, HER2 amplified Luminal -like; HER2 nonLu like, HER2 amplified non luminal-like; BTAMs, Breast adipose tissue macrophages; BTSMs, Breast tumor-stroma macrophages; HR hazard ratio, CI, confidence interval; *, significant (p-value < 0.05).
